# Meeting of the Medical Association of Georgia

**Published:** 1904-05

**Authors:** 


### *EDITORIAL NOTES AND COMMENTS.*

The Business office of THE JOURNAL-RECORD is No. 1007-1008 Century Building.

The Editorial office is 318-319-320 The Prudential.

Address all Business communications to Dr. M. B. Hutchins, Mgr.

Make remittances payable to THE ATLANTA JOURNAL-RECORD OF MEDICINE.

On matters pertaining to the Editorial and Original Communications address Dr. Bernard Wolff, Atlanta.

Reprints of original articles will be furnished at cost price. Requests for the same should always be made *on the manuscript.*

We will present, post-paid, on request, to each contributor of an original article, twenty (20) marked copies of THE JOURNAL-RECORD containing such article.

### MEETING OF THE MEDICAL ASSOCIATION OF GEORGIA.

THE fifty-fifth annual meeting of the Georgia Medical Association was held at Macon April 20–22.

The copy of the program appended shows a long list of papers, many of which were actually read and discussed, while many others were, in the absence of the authors, presented by title and will appear in the Transactions.

The attendance was good and quite a number of new names were enrolled.

There was one notable feature of the meeting scarcely commendable, and that was the amount of valuable time consumed in windy debates of a quasi-political character. This is not entirely fair to those who come to read papers and who confidently expect to find an attentive, appreciative audience. Instead they are very apt to find their places on the program dislocated beyond ready reposition, and when at length opportunity presents for them to deliver themselves they find their labor is vain and their words beat upon dull ears and preoccupied brains.

It might not be an altogether idle suggestion for those who regard the meetings as a species of political caucus to hire a tent for themselves and allow those who entertain other views on the function of the gathering to read their papers in single-minded tranquility.

It is a cruel disappointment for a man to prepare a paper months in advance, picturing to himself a large concourse of listeners interested and appreciative of his efforts, only to find at the supreme moment that political matters have so engrossed the attention of his audience that the aroma has evaporated from his theme and that it falls as flat and pale and featureless as a length of tapeworm.

Upon such a one the impression of the meeting is so disagreeable that he is not disposed to invite another snub by a repetition of his indiscretion in attending the meetings.

There should be a definite time allotted for the executive sessions and the discussion of the various questions pertaining to it, and another time devoted to the reading of papers and the two should in no way conflict or overlap.

The scheme for adoption of the plan of organization outlined by the American Medical Association through its accredited representative, Dr. J. M. McCormick, of Kentucky, was the most important matter up for discussion. Nothing definite was accomplished, however, as the motion to adopt the plan with some minor changes was tabled until next year. Tabling the motion was in accord with the desire to have more time for an appreciative and deliberate study of what the plan of organization involved, and especially its effect upon the autonomy and integrity of the State Association. It is presumed that each member, as is customary, will at once diligently set about this work and that when the matter is again brought up there will be as many solutions of the problem as there were dialects at Babel. The tuberculosis question was much discussed and many suggestions and recommendations were made as touching its management and prevention of its spread. Dr. George Brown spoke of the work of the American Anti-Tuberculosis League, and also recommended State care of the indigent tuberculous. The important matter of State Board of Medical Examiners reciprocity was up and a committee was appointed to take it in hand. Many groups of States have now arranged a basis of recognition of liscense and it looks as though the movement would become general.

A disagreable episode was the suspension, for five years, by the Board of Censors, of a member whose paper published in the last year's volume of the Transactions, bore such a startling resemblance to some of the writings of Dr. G. Frank Lydston, of Chicago, as to call forth from the latter something which might be called by those who are not overly nice in the choice of words, a protest. Neither were the Censors pleased at this alleged adaptation, and resented it by the sequestration of the offender.

The social side of the meeting was well looked after by the local members and well enjoyed by the visitors.

The Macon meetings are always pleasant, especially to the Atlanta contingent, who find in its rural tranquility and the cloistral stillness of its streets an agreeable change from the hurly-burly of a great city.

PROGRAM.

WEDNESDAY, APRIL 20.—Morning Session, 9:30 O'clock.

Prayer by Rev. R. E. Douglas.

Address of Welcome in behalf of the city. Mayor Bridges Smith.

Address of Welcome in behalf of the citizens. Hon. Minter Wimberly.

Address of Welcome in behalf of local profession. Dr. Howard J. Williams.

Response to Addresses of Welcome. Dr. Wm. Perrin Nicolson.

Report of Committee on Arrangements.

Report of Secretary and Treasurer.

Filling vacancies on Board of Censors and Executive Committee.

Applications for membership.

1. The Treatment of Cancer. M. B. Hutchins, M.D., Atlanta.

2. The Moral and Religious Side of the Medical Profession. E. Morgan, M.D., Vidalia.

3. Some Dangers to the Public Health of Georgia Needing Control by Legislation. R. M. Harbin, M.D., Rome.

4. A Plea for More Thorough Examination and Rational Treatment of Nose and Throat Disease. Dunbar Roy, M.D., Atlanta.

5. Two Cases of Ectopic Pregnancy Going to Term. E. R. Corson and J. G. Van Marten, M.D., Savannah.

6. Ectopic Gestation, with Report of Complete Operation, with Recovery of Patient. E. C. Davis, M.D., Atlanta.

7. A Few Surgical Cases. O. T. Kenyon, M.D., Dawson.

8. The Clinical Consideration of Tumors. W. F. Westmoreland, M.D., Atlanta.

9. Consumption and The State. M. M. Saliba, M.D., Savannah.

10. Adenoids—Their Complications and Treatment. J. L. Hiers, M.D., Savannah.

AFTERNOON SESSION, 2:30 O'CLOCK.

Report of Board of Censors.

Application for membership.

11. Renal Decapsulation—Indications, Limitations and Technique. R. R. Kime, M.D., Atlanta.

12. Hysterectomy for Fibroid Tumors of the Uterus. Geo. H. Noble, M.D., Atlanta.

13. Forty-two Consecutive Laparotomies in the Practice of a Country Doctor, and Report of Cases. F. M. Ridley, M.D., LaGrange.

14. Some Frequently Misunderstood Symptoms of General Interest in the Region of the Head. A. W. Stirling, M.D., Atlanta.

15. The Prophylaxis of Venereal Diseases. W. L. Champion, M.D., Atlanta.

16. When to Circumcise the Infant if Not on the Eighth Day. S. A. Visanska, M.D., Atlanta.

17. Anesthesia and Anesthetists. R. M. Thomson, M.D., Savannah.

18. Nitrous Oxide Gas and Ether Anæsthesia. H. P. Derry, M.D., Macon.

19. Uncinariasis in Georgia. Claude A. Smith, M.D., Atlanta.

20. Treatment of Enlargement of the Prostate. Geo. R. White, M.D., Savannah.

21. The Treatment of Prostatitis with the Wave Current from a Static Machine. R. P. Izlar, M.D., Waycross.

22. Pneumonia as Influenced by Senility and Alcoholism. J. E. New, M.D., Dexter.

23. It's Only a Running Ear. J. M. Crawford, M.D., Atlanta.

24. Clinic of Deaf Mutes, whose Hearing and Speech Have Been Established and the Instrument and Technic Applicable to Various Diseases and Deaf Conditions. M. M. Stapler, M.D., Macon.

THURSDAY, APRIL 21.—Morning Session, 9 O'clock.

Reading of Minutes.

Report of Board of Censors.

Report of Executive Committee.

Application for membership.

25. The Best Operation for Vesical Calculus, with Report of Cases. M. F. Carson, M.D., Griffin.

26. The Specific Treatment of Morphinism. C. C. Stockard, M.D., Atlanta.

27. Incurable Headache—Report of Two Cases. V. D. Lockhart, M.D., Maysville.

28. Tuberculosis. J. E. Sommerfield, M.D., Atlanta.

29. The Prevention of Tuberculosis. T. E. Oertel, M.D., Augusta.

30. The Prevention of Tuberculosis. George Brown, M.D., Atlanta.

31. The Phenomena of a Dream. W. A. O'Daniel, M.D., Bullards.

32. The Instantaneous Cure of Erysipelas. W. B. Taylor, M.D., Dexter.

33. Treatment of Ulcers. A. L. Fowler, M.D., Atlanta.

34. The Necessity of a State Board of Examiners for Trained Nurses for Georgia. E. B. Elder, M.D., Macon.

35. Eye Strain—the Importance of its Detection and Correction in all General Nervous Derangements. A. G. Hobbs, M.D., Atlanta.

36. The Association of Cataract with Uncinariasis or Hook-Worm Disease. A. W. Calhoun, M.D., Atlanta.

37. Four Interesting Cases of Kidney Surgery. Thos. R. Wright, M.D., Augusta.

38. How to Make Thin People Fat, or How to Make Weak People Strong. J. C. McAfee, M.D., Macon.

THURSDAY, 2 P.M.—Barbecue at the Acme Brewing Company.

THURSDAY NIGHT, 8 O'CLOCK.

39. Antiseptic and Eliminative Treatment of Typhoid Fever. T. V. Hubbard, M.D., Atlanta.

40. The Rational Treatment of Tabetic Ataxia. Theodore Toepel, M.D., Atlanta.

41. Report of a Case of Twins of Unequal Size and Age. W. W. Evans, M.D., Higgston.

42. A Plea for More Careful Abdominal Diagnosis. W. P. Nicolson, M.D., Atlanta.

43. Intestinal Obstruction. C. T. Nolan, M.D., Marietta.

44. The Antiseptic and Eliminative Treatment of Typhoid Fever, with Clinical Report. W. E. Fitch, M.D., Savannah.

45. Smallpox, with Especial Reference to the Extraordinary Mild Epidemic of this Disease now Prevailing in Georgia. H. F. Harris, M.D., Atlanta.

46. Creosotal in the Treatment of Pneumonia, with Report of Case. J. D. Herrman, M.D., Eastman.

FRIDAY, APRIL 22.—Morning Session, 9 O'clock.

Reading of Minutes.

Report of Board of Censors.

Application for membership.

Report of Standing and Special Committees.

47. Pneumonia and its Treatment. H. R. Slack, M.D., LaGrange.

48. Appendicitis—the Limitations of Medical Treatment—the Indications for Surgical Interference. F. W. McRae, M.D., Atlanta.

49. The Philosophy of Suggestion—a Theory. Jno. R. Rose, M.D., Eastman.

50. Influenza and its Attending Complications. W. S. Kendrick, M.D., Atlanta.

51. The Simplest and Best Leg Holder; the Simplest and Best Anesthetic Dropper. T. M. McIntosh, M.D., Thomasville.

52. The Hyperdermic Administration of Magnesium Sulphate. J. R. Garner, M.D., Atlanta.

53. The Study of Floating Kidney, with its Treatment. W. M. Smith, M D., Atlanta.

54. Hernia in its Relation to Trauma. J. B. Morgan, M.D., Augusta.

AFTERNOON SESSION, 3 O'CLOCK.

55. Hemorrhagic Typhoid in a Child—Recovery. M. McH. Hull, M.D., Atlanta.

56. Differentiation Between Typhoid and Malarial Fevers, with Treatment of Both. Chas. Hicks, M.D., Dublin.

57. A Plea for More Thorough Preliminary Education of the Student, Before Entering Upon the Study of Medicine. A. A. Smith, M.D., Hawkinsville.

58. Hydrorrhea Gravidarum. Alexander Mack, M.D., Hawkinsville.

59. Cases of Genu-Valgum and Genu-Varum—Operated. W. J. Little, M.D., Macon.

60. Chronic Catarrh of the Stomach. J. N. LeConte, M.D., Atlanta.

61. Echinacea; Some of its Uses in Modern Surgery. A. B. Mathews, M.D., Elberton.

62. Victims of the New Holland Cyclone, with Report of Six Special Cases. Drs. J. H. Downey and H. L. Rudolph, Gainesville.

---

THE following additions to the Presbyterian Hospital Staff have been made with the title of Associate: Internal Medicine, J. N. LeConte; Gynecology, W. B. Sutton; Genito-Urinary Diseases, Bernard Wolff.

---

THE Grady Hospital has a new operating-room in process of construction.

